# Metabolic Control of Stemness and Differentiation

**DOI:** 10.1155/2019/6865956

**Published:** 2019-06-12

**Authors:** Viviana Moresi, Athanassia Sotiropoulos, Giuseppina Caretti, Alexandra Harvey

**Affiliations:** ^1^Department of Anatomy, Histology, Forensic Medicine & Orthopedics, Histology & Medical Embryology Section, Sapienza University of Rome and Interuniversity Institute of Myology, Italy; ^2^Centre National de la Recherche Scientifique UMR 8104, Institut Cochin, Université Paris Descartes, Sorbonne-Paris-Cité, Paris, France; ^3^Department of Biosciences, Università degli Studi Milano, Milan, Italy; ^4^School of BioSciences, University of Melbourne, Parkville VIC, Australia

Increasing evidence highlights a pivotal role for metabolism in stem cell physiology and lineage specification [1, 2]. Metabolism, indeed, is no longer considered merely an energy source nor an endpoint of gene regulation. Instead, metabolites and the nutrient environment are active players in determining intracellular signaling and enzymatic activities and consequently modulators of stem cell fate. Moreover, metabolic intermediates of cellular metabolism regulate epigenetic mechanisms, including histone modifications, DNA methylation, and noncoding RNAs, thereby modulating the global epigenome landscape and stemness [3].

This special issue brings together 9 papers to highlight recent developments in the field.

The most recent studies addressing the relevance of metabolism and nutrient availability in regulating stem cell biology are expertly reviewed. S. Wu et al. review the importance of one-carbon metabolism in mediating epigenetic modifications, i.e., DNA methylation, histone modification, and microRNAs, during embryonic development. Further, J. Spyrou et al. reviewed the critical role of the microenvironment in the retention of somatic cell memory by induced pluripotent stem cells, identifying previously poorly documented areas in this field. Lastly, C. Xie et al. review the emerging and debated role of mesenchymal stem cells in the vascular calcification process, reporting studies that support a role in facilitating vascular calcification, while others declare a protective role of mesenchymal stem cells in this process. Both these conflicting theories imply the modulations of cell-cell communications, paracrine signals, and exosomes and of the vascular microenvironment (vessel niche) that will impact cell fate.

In an extension of the role of metabolism in regulating stemness, W. Fu et al. and L. Papa et al. review the crucial role of mitochondrial dynamics in stem cell behavior and reprogramming. Changes in mitochondrial activity are no longer considered a passive consequence of metabolic reprogramming from glycolysis to oxidative phosphorylation during stem cell differentiation. Rather, the modulation of mitochondrial activity is critical for programming stem cell fate and differentiation potential [4, 5]. W. Fu et al. summarize the regulation of mitochondrial dynamics by intrinsic, extrinsic, and pharmacological factors and the impact of mitochondrial dynamics on stem cell fate, defining potential new applications in stem cell-based therapy. L. Papa et al. highlight recent advances in the emergent role of mitochondria in hematopoiesis, where the modulation of mitochondrial activity is critical for maintaining hematopoietic stem cell self-renewal potential and may be considered a tool to increase the *ex vivo* expansion of transplantable cells.

Given the strong link between metabolism and heritable changes to the epigenetic landscape, casting light on the mechanisms underlying the metabolic regulation of stem cell self-renewal and differentiation is important not only to understand tissue homeostasis in both physiological and pathological conditions but also to establish *in vitro* culture conditions which accurately replicate the physiological microenvironment to support normal cell physiology and function. To this end, J. G. Lees et al. elucidate the impact of physiological oxygen levels (5%) on human embryonic stem cells by integrating metabolic, transcriptomic, and epigenetic analyses. J. G. Lees et al. report that 5% oxygen increases glycolytic intermediates, glycogen, and the antioxidant response; reduces mitochondrial metabolism; and establishes a more permissive structure global epigenetic landscape in embryonic stem cells.

Nutrient availability also directly regulates the progression from a quiescent to an activated state of MuSC [6]. Muscle stem cells (MuSC) display low oxygen consumption and mitochondrial activity and are characterized by a specific fatty acid oxidation profile in their quiescent state. When activated, they increase glycolysis, fatty acid metabolism, and oxidative phosphorylation. Short-term caloric restriction has been shown to favor MuSC self-renewal, increasing oxidative activity and decreasing glycolysis [7]. In this special issue, T. Pavlidou et al. demonstrate that treatment with metformin, a calorie-restriction mimicking drug, *in vitro*, *ex vivo*, or *in vivo*, favors MuSC quiescence and a low metabolic state, delaying their activation and skeletal muscle regeneration.

Different metabolic features are often a signature of distinctive stem cell subpopulations. In this special issue, through high-resolution nuclear magnetic resonance metabolomic analyses of cell supernatants, C. Lefevre et al. define distinct metabolic signatures between visceral and subcutaneous adipose tissue stem cells, differentiated by their requirement for glutaminolysis and their ability to utilize pyruvate. These divergent metabolic profiles may contribute to the differing abilities of these populations to proliferate and to differentiate into adipocytes.

Alterations in metabolism have been associated with, and may contribute to, the onset or progression of numerous diseases. In support of this, A. Bordin et al. demonstrate a clear and biologically relevant effect of type 2 diabetes (T2D) patients' oral plaque in negatively regulating dental pulp stem cell clonogenicity. Their data suggest that there are key differences in plaque from T2D patients with periodontal disease (PD) relative to healthy individuals with PD which may be a result of underlying metabolic changes.

Collectively, the studies in this special issue provide insights into the metabolic control of stem cell maintenance, differentiation, and engraftment and also provide evidence for novel pharmacological interventions to manipulate stem cells. In addition, by elucidating the mechanisms underlying stem cell biology and highlighting the connections between cellular metabolism, mitochondria, and epigenetic regulation of stem cell fate, this special issue will have important implications for advances in stem cell research and reprogramming, particularly regarding novel approaches in regenerative medicine.
